# Molecular Mechanisms and Therapeutic Role of Intra-Articular Hyaluronic Acid in Osteoarthritis: A Precision Medicine Perspective

**DOI:** 10.3390/jcm14082547

**Published:** 2025-04-08

**Authors:** Wojciech Glinkowski, Dariusz Śladowski, Wiesław Tomaszewski, Pol-IAHA Study Group

**Affiliations:** 1 Center of Excellence “TeleOrto” for Telediagnostics and Treatment of Disorders and Injuries of the Locomotor System, Department of Medical Informatics and Telemedicine, Medical University of Warsaw, 02-091 Warsaw, Poland; 2 Stichting Med Partners, 1098 XH Amsterdam, The Netherlands; 3 Department of Transplantology and Central Tissue Bank, Medical University of Warsaw, 02-004 Warsaw, Poland; 4 ARS MEDICA Foundation for Medical Education, Health Promotion, Art and Culture, 03-301 Warsaw, Poland; 5 College of Physiotherapy, 50-038 Wrocław, Poland

**Keywords:** osteoarthritis, hyaluronic acid, viscosupplementation, molecular mechanisms, chondroprotection, anti-inflammatory, molecular weight, pain modulation, precision medicine

## Abstract

**Background**: Osteoarthritis (OA) is a degenerative joint disease characterized by progressive cartilage breakdown, synovial inflammation, and pain, which leads to significant disability. IAHA is widely used because of its viscoelastic properties, which restore synovial fluid homeostasis and reduce symptoms. However, emerging evidence suggests that IAHA exerts additional biological effects including chondroprotection, inflammatory modulation, oxidative stress reduction, and pain modulation, which may influence disease progression. **Objective**: This narrative review examines the biological mechanisms underlying IAHA’s role in OA management. The review explored IAHA’s effects on synovial fluid viscoelasticity, inflammatory cytokine modulation, cartilage preservation, oxidative stress regulation, and pain pathways, emphasizing the influence of molecular weight variations on therapeutic efficacy. Additionally, this review evaluates IAHA’s integration into multimodal treatment strategies, its potential disease-modifying effects, and future directions for personalized treatment approaches. **Methods**: A comprehensive literature review was conducted using PubMed, Cochrane Library, EMBASE, Scopus, and Web of Science for studies published between January 2000 and March 2024. The search focused on IAHA’s molecular, cellular, and biochemical effects in OA and clinical findings assessing its impact on joint function, pain relief, and disease progression. **Results**: IAHA improves synovial fluid lubrication, reduces proinflammatory cytokines (IL-1β, TNF-α), inhibits matrix metalloproteinases (MMPs), scavenges reactive oxygen species (ROS), and modulates nociceptive pathways. High-molecular-weight IAHA demonstrates superior efficacy in advanced OA, while low-molecular-weight formulations may be better suited for early-stage disease. Although IAHA’s symptom relief is comparable to corticosteroids and NSAIDs, its favorable safety profile and emerging disease-modifying potential support its long-term use in OA management. **Conclusions**: IAHA represents a multifaceted therapeutic approach bridging symptomatic relief and regenerative strategies. While long-term efficacy, optimal administration protocols, and patient-specific responses remain subjects of ongoing research, refining treatment selection criteria, dosing regimens, and combination strategies may enhance clinical outcomes. Future studies should explore biomarker-driven approaches, standardize treatment protocols, and assess IAHA’s synergy with regenerative medicine to optimize its role in OA management.

## 1. Introduction

Osteoarthritis (OA) is one of the most prevalent chronic joint diseases, leading to progressive cartilage degeneration, inflammation, and pain, with a significant impact on quality of life. OA, the most prevalent form of arthritis worldwide [1,2,3,4], affects a substantial proportion of the aging population [2,5,6,7,8]. The incidence of OA is higher than that of other arthropathies [1,2], and according to the World Health Organization (WHO) [9], up to 25% of the population aged >65 years may require treatment, including surgery, for OA [4,10]. In 2020 [2], an estimated 595 million people worldwide were affected by OA, representing 7.6% of the global population [2,11]. OA has increased by 132.2% since 1990, and by 2050, the number of cases is expected to rise significantly across various joints, including the knee, hand, and hip [7,12,13,14]. OA is characterized by the gradual deterioration of the joints, including the destruction of joint cartilage [1,15,16,17], leading to joint pain [2,12], stiffness, swelling, and reduced mobility [6]. The development of OA results from a complex interplay of mechanical stress (obesity), genetic predisposition, metabolic factors, aging, and inflammatory processes that contribute to cartilage breakdown and changes in the subchondral bone [18,19,20]. In addition to articular cartilage, OA involves the subchondral and trabecular bones and all other joint structures. Exposed subchondral bone increases pain and reduces joint mobility during cartilage decay. OA can affect any joint, but the most commonly affected joints bear the body weight and are highly susceptible to mechanical stress [1,2,5,21]. OA can present as an asymptomatic incidental finding or a severely disabling disorder [1,4,11,18,19]. Progressive degeneration of articular cartilage reduces the structural integrity of the joint and significantly deteriorates the quality of life. OA is a significant public health problem because of its prevalence and significant impact on mobility and activities of daily living (ADL) [22]. Traditionally, OA is categorized into two major forms: Primary OA (Idiopathic) and Secondary OA. Primary OA occurs without any identifiable underlying cause, while Secondary OA develops in the presence of a specific predisposing factor or pathology (e.g., congenital abnormalities, trauma, or avascular necrosis). Primary or idiopathic OA arises without obvious traumatic, congenital, or metabolic triggers. It is most strongly associated with aging and the cumulative effects of mechanical loading over time. Common sites involved in osteoarthritis include the knees, which are often affected due to their critical role in weight-bearing and mobility; the hips, where degenerative changes in the femoral head and acetabulum cause significant disability; the hands, particularly the distal interphalangeal joints (Heberden’s nodes) and proximal interphalangeal joints (Bouchard’s nodes); the spine, with the cervical and lumbar regions frequently involved, leading to facet joint OA; the first carpometacarpal joint (base of the thumb), which contributes to grip and pinch strength, with OA here causing functional limitations; and the first metatarsophalangeal joint (hallux rigidus), which manifests as pain and stiffness in the big toe. Secondary OA develops when a known pathological or mechanical factor contributes to the breakdown of normal joint architecture. Key causes include congenital abnormalities, traumatic injuries, and conditions that compromise the structural integrity of bone and/or cartilage. Post-traumatic OA arises when an injury (acute or chronic) disrupts normal joint structure. Even with repair or reconstruction, joint integrity may not fully return to its pre-injury condition, setting the stage for progressive cartilage degeneration.

Avascular necrosis, also known as osteonecrosis, entails the loss of blood supply to the subchondral bone. It is most common in the femoral head but can affect the humeral head, talus, scaphoid, or other bones. Secondary forms have a well-defined primary pathogenetic factor that should be prioritized for treatment [23,24]. In contrast, primary OA is influenced by its localization, which is linked to a distinct pathogenetic factor. As a result, different therapeutic interventions may have varying effects. Endophenotypes refer to distinct subgroups of patients within the exact localization of primary OA, categorized based on specific clinical, biological, or morphological characteristics. In recent years, scientists and health professionals have focused on identifying and characterizing the phenotypes of OA and its associated endotypes. As a starting point, six clinical phenotypes and nine endotypes of knee OA have been described [25]. Four distinct endophenotypes were identified for hip OA (HOA) [26], each reflecting specific clinical, radiological, or biochemical patterns. These variations highlight the multifaceted nature of HOA, in which factors such as joint congruence, acetabular morphology, and systemic inflammatory markers intersect differently in individual patients. By contrast, the categorization of hand OA remains a subject of ongoing debate. While [26] proposed three endophenotypes, ref. [27] suggested five, reflecting diverse presentations involving different joints in the hand, inflammation levels, and cartilage degeneration patterns. These discrepancies underscore the complexity of OA pathogenesis and the need for continued research. These endophenotypes help clarify why certain patients may present with more pronounced inflammatory changes, whereas others exhibit primarily mechanical issues or cartilage deterioration. By identifying and understanding these subgroups, clinicians can better tailor treatment approaches, such as targeted pharmacotherapy for inflammation, mechanical realignment procedures, regenerative techniques, and other interventions, aiming for more effective and personalized management of OA. Although widely used, IAHA is often seen as merely symptomatic rather than disease-modifying.

IAHA has a long history, originating in the early 1990s as a way to restore rheological balance within the joint [27,28]. Early research highlighted IAHA’s ability to supplement synovial fluid and improve lubrication. Over time, multiple clinical studies supported its efficacy in alleviating pain and enhancing joint function, incorporating it into numerous treatment guidelines [22,29,30]. As a result, IAHA has become a commonly employed intervention for osteoarthritis management, particularly in knee OA. Meanwhile, researchers continue investigating novel formulations, varying molecular weights, and adjunctive therapies to optimize outcomes, minimize side effects, and address challenges such as patient selection and long-term efficacy. This review demonstrates that IAHA remains a dynamic and evolving component of osteoarthritis treatment.

Recent insights into IAHA’s molecular and cellular mechanisms of IAHA, particularly its anti-inflammatory, chondroprotective, and oxidative stress-modulating effects, suggest its broader role in OA management. These findings and evidence of tailored applications based on molecular weight and disease stage warrant further investigation.

### Hyaluronic Acid (HA) in OA Management

Despite numerous treatment options, including non-pharmacological and pharmacological interventions, there is an urgent need for more effective long-term strategies to alleviate symptoms and delay surgical intervention. HA, a naturally occurring glycosaminoglycan in synovial fluid, is vital for joint homeostasis [31,32]. It enhances the viscoelastic properties of synovial fluid, which are crucial for joint lubrication, shock absorption, and smooth, pain-free movement [33,34,35,36,37,38]. In OA, particularly KOA, HA concentration and molecular weight in synovial fluid are often reduced, leading to decreased lubrication and increased joint friction, exacerbating symptoms, and accelerating degeneration [34]. Viscosupplementation (VS) involving IAHA has become an essential treatment option [39,40,41,42,43,44,45,46,47,48,49,50,51,52], a process in which it is directly injected into the affected joint [49,50,53,54,55,56,57,58,59,60,61,62,63,64,65]. IAHA aims to restore the viscoelastic properties of synovial fluid, reduce pain, and improve joint function, potentially delaying surgical interventions, such as total joint replacement [66,67,68,69,70,71].

This review examines the modulation of key biological processes in osteoarthritis (OA) by intra-articular hyaluronic acid (IAHA), including synovial fluid homeostasis, chondroprotection, inflammatory suppression, oxidative stress reduction, and pain modulation. It synthesizes current evidence regarding IAHA’s clinical effectiveness in OA management, focusing on molecular weight variations. The review assesses IAHA’s potential disease-modifying effects, its role in multimodal treatment strategies, and future biomarker-driven personalized approaches. Identifying research gaps aims to provide insights for optimizing IAHA therapy in clinical practice. The study aimed to collate and discuss IAHA’s mechanisms and clinical aspects from various studies, rather than pool statistical results into a single quantitative effect size estimate. Many included studies use different populations, outcome measures, and interventions (such as varying IAHA formulations), making direct comparisons or pooled statistical analyses challenging. Consequently, this work focuses on synthesizing themes and identifying mechanistic insights, rather than combining datasets for inferential statistics.

## 2. Materials and Methods

### 2.1. Literature Search and Data Sources

This narrative review synthesizes the current evidence of the biological mechanisms of IAHA in osteoarthritis (OA). A literature search was conducted across PubMed, the Cochrane Library, EMBASE, Scopus, and Web of Science for studies published between January 2000 and December 2024. The key mechanisms of action of IAHA in OA treatment have been investigated and analyzed. Multiple mechanisms comprising the potential joint effects were traced, and the following were highlighted: viscoelastic and lubricating action, chondroprotection, modulation of inflammation, reduction in oxidative stress, and modulation of pain. The search terms were combined using Boolean operators (AND and OR). Core IAHA concepts keywords included the following: “hyaluronic acid” OR “hyaluronan” OR “IAHA” OR “viscosupplementation”; Osteoarthritis Pathophysiology: “osteoarthritis” OR “degenerative joint disease” OR “cartilage degradation”; Biological Mechanisms: “synovial fluid homeostasis” OR “joint lubrication” OR “viscoelasticity”; Chondroprotection: “cartilage preservation” OR “proteoglycan synthesis” OR “extracellular matrix stability”; Inflammatory Modulation: “cytokines” OR “immune response” OR “CD44 receptor” OR “RHAMM receptor”; Oxidative Stress Regulation: “reactive oxygen species” OR “antioxidant defense” OR “oxidative damage”; Pain Modulation: “nociceptive pathways” OR “neuroinflammation” OR “pain signaling”; Molecular Weight Considerations: “low molecular weight HA” OR “high molecular weight HA”. Additional studies were identified by manually screening the reference lists from systematic reviews and meta-analyses.

### 2.2. Criteria Selection Criteria for the Study

Studies were selected based on predefined criteria:

Inclusion Criteria:

The inclusion criteria encompassed randomized controlled trials (RCTs), observational cohort studies, systematic reviews, and investigations into the molecular mechanisms of intra-articular hyaluronic acid (IAHA) in synovial fluid and cartilage. Studies were selected based on mechanistic investigations exploring the effects of IAHA; assessments of IAHA’s impact on synovial fluid, cartilage, inflammation, oxidative stress, and pain pathways; comparative analyses of IAHA formulations based on molecular weight; and evaluations of IAHA’s potential as a disease-modifying agent. Only articles published in English were considered for inclusion in the present study. Broad inclusion criteria were employed because the primary objective was to delineate the clinical and mechanistic landscape of IAHA. The study included peer-reviewed original research articles, reviews, and meta-analyses focusing on IAHA or viscosupplementation in human osteoarthritis (OA) populations, as well as studies providing mechanistic insights into the effects of hyaluronic acid on cartilage, synovial fluid, inflammation, oxidative stress, or pain modulation.

Exclusion Criteria:

Studies that lacked clinical relevance to humans and those without molecular or biochemical evaluations were excluded. Strategies such as sensitivity analysis by excluding low-quality studies, prioritizing studies from high-impact journals, categorizing results by study design to highlight discrepancies, and critically evaluating bias in industry-sponsored studies were employed to address conflicts arising from contradictory studies. Non-peer-reviewed articles, conference abstracts, case reports, and animal or in vitro studies without clinical relevance were excluded. Furthermore, studies focusing solely on the efficacy and safety of IAHA, without discussing the biological mechanisms of action, were not considered. The exclusion criteria included conference abstracts lacking sufficient data for assessment, animal or in vitro studies that did not provide clinically translatable insights, and articles unrelated to intra-articular HA or OA pathophysiology.

### 2.3. Data Extraction and Synthesis

Two independent reviewers extracted data, including study details (author, year, journal), study design (RCT, cohort study, mechanistic study, review), patient characteristics (OA severity, synovial fluid composition, prior treatments), intervention details (IAHA molecular weight, dosing regimen, and frequency), and mechanistic outcomes (synovial fluid restoration and lubrication, chondroprotection and extracellular matrix integrity, inflammatory cytokine suppression, oxidative stress reduction, and pain pathway modulation).

Discrepancies were resolved by consensus discussion, and a third reviewer was consulted if necessary. Because of the heterogeneity in the study designs, IAHA formulations, and mechanistic endpoints, a narrative approach was used.

## 3. Results

**Figure 1:**
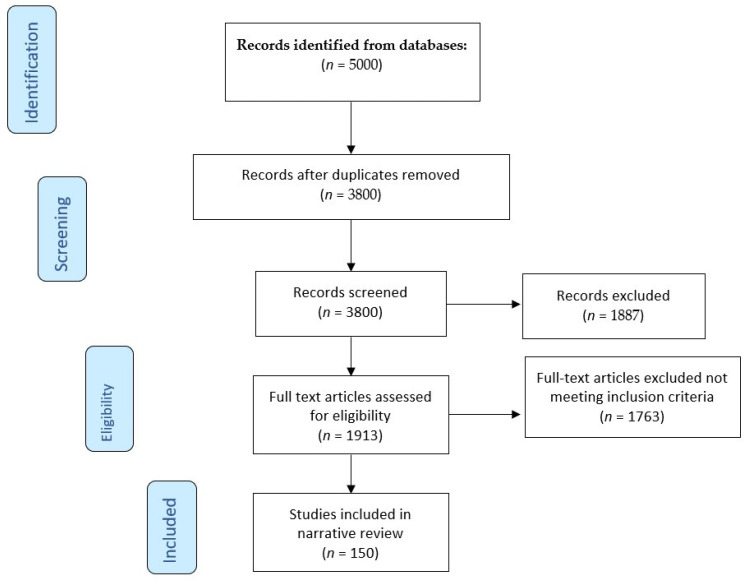
Flow chart of the literature search using PRISMA (Preferred Reporting Items for Systematic Reviews and Meta-Analyses).

The review used the PRISMA guidelines’ rigorous and transparent methodological approach (Figure 1).

### 3.1. Hyaluronic Acid

Hyaluronic acid (HA), hyaluronan, and hyaluronate are critical in managing OA, particularly knee OA [31,32]. Its application in VS aims to restore the viscoelastic properties of synovial fluid, alleviate pain, improve joint function, and potentially delay surgical intervention [33,34,35,36]. The pathophysiology of OA involves mechanical joint stress, genetic susceptibility, ethnicity, nutrition, sex [35], and age-related alterations in HA concentration and molecular weight within the joint tissues [34].

HA is a non-sulfated glycosaminoglycan (GAG) mucopolysaccharide, an anionic polysaccharide found in all living organisms [72]. It was initially isolated from a bovine vitreous body and reported in 1934 by Karl Meyer and John W. Palmer [73]. HA’s first biomedical application of HA for ophthalmic surgery occurred in the late 1950s [74]. Its chemical structure and biological properties have led to its extensive utilization in therapies such as dermal fillers, tissue engineering, VS, ocular treatments, wound healing, drug delivery, and cancer therapy [36,72]. Intra-articular injections of VS have been documented in various joints, including the knee, hip, shoulder, ankle, temporomandibular, and interphalangeal joints. Currently, HA is indicated for OA treatment in any synovial joint, either as part of a comprehensive treatment plan or selectively, particularly in early-stage OA.

### 3.2. Hyaluronan (HA) Production

Hyaluronan (HA) is a polysaccharide of significant biological importance and is synthesized by specialized enzymes known as hyaluronan synthases, which reside on the cytoplasmic face of the plasma membrane [75]. These enzymes meticulously incorporate UDP-activated sugar residues during polymerization into an elongated HA chain. The newly formed hyaluronan strand is then carefully moved through channel-like or pore-like structures, ensuring its final destination is outside the cell in the extracellular compartment [76].

Mammals have three primary hyaluronan synthases: HAS1, HAS2, and HAS3 [77,78,79,80]. Although each has a distinct gene locus, all three enzymes share a fundamental enzymatic characteristic; they use two specific substrates, UDP-α-N-acetyl-D-glucosamine (UDP-GlcNAc) and UDP-α-D-glucuronate (UDP-GlcUA), to build the HA chain [81]. Several researchers have categorized known HA synthase enzymes into Class I and II based on their homology, catalytic features, and evolutionary lineage. The eukaryotic and Streptococcal HAS isoforms align with Class I, whereas a lone representative from *Pasteurella multocida* comprises Class II [82]. This classification has helped clarify functional and structural differences among synthases from diverse organisms. A substantial portion of our understanding of HA synthase’s mechanistic intricacies comes from bacterial HA synthase [83,84]. A significant leap forward in the field has occurred with the successful cloning of *HAS* genes from both prokaryotic and mammalian organisms. The DeAngelis group notably isolated and characterized the *hasA* gene from Group A *Streptococcus*, which encodes a protein called HasA. Subsequent studies confirmed that HasA is a bona fide HA synthase in *Streptococcus pyogenes*. Moreover, transferring HasA into bacteria lacking native HA capsules, such as certain *Streptococcus* strains and *Enterococcus faecalis*, enables these organisms to synthesize HA, thereby establishing HasA as the core enzyme in forming the bacterial HA capsule [82]. Parallel research efforts in mammals have led to the cloning of mouse and human HA synthases [85]. Upon further examination, these mammalian enzymes showed a high degree of amino acid sequence homology between themselves and across various species, including frogs (*Xenopus*) and certain bacteria. Notably, the human haS1 isoform closely resembles the *hasA* gene product of *Streptococcus pyogenes*, highlighting a striking level of evolutionary conservation. Additional work has mapped the genomic locations and structural organization of *HAS1*, *HAS2*, and *HAS3*, revealing that they occupy different autosomes. These findings suggest that the *HAS* gene family emerged early in vertebrate evolution, possibly via the serial duplication of an ancestral *HAS* gene. Collectively, these insights illuminate the complex molecular choreography of HA biosynthesis, from genetic foundations and enzyme architecture to stepwise addition of sugar units and extracellular release of the growing polysaccharide. By integrating bacterial and eukaryotic findings, researchers have shed light on how a widely conserved enzyme family orchestrates the formation of this critical extracellular matrix component, which is indispensable to both health and disease. Most HA supplementation products are avian-derived; hyaluronan is often sourced from rooster combs and then chemically crosslinked or produced via bacterial fermentation [74]. While many fermentation protocols employ genetic engineering to increase yield or alter properties, these items are seldom labeled “recombinant”. Instead, manufacturers typically say “fermented HA” or “microbial fermentation”, even if the process could be considered recombinant in a technical sense. Both avian and bacterial sources of HA require thorough purification. Residues from either process may pose side effect risks if they are not removed. In peer-reviewed studies on hyaluronic acid (e.g., for viscosupplementation injections), the origin of HA is often specified, although not always standardized. Consequently, physicians and patients may be unaware of the differences among the various HA products in everyday clinical use.

### 3.3. Molecular Mechanisms of IAHA

HA, a naturally occurring substance in the body, exerts therapeutic effects on OA via several physical and molecular mechanisms. These mechanisms are crucial for understanding HA’s role in OA [86].

HA restores the flexibility of synovial fluid and improves joint movement and shock absorption [87]. This is primarily achieved by increasing the concentration and size of hyaluronic acid molecules in the joint. At the molecular level, hyaluronic acid is a large molecule with repeating sugar units. When injected into the joint, hyaluronic acid supplements the naturally occurring hyaluronic acid, increasing its size and concentration in the synovial fluid [34,87,88]. The electrostatic and osmotic properties of the extracellular matrix (ECM) are also improved, thanks to proteoglycans that contain negatively charged glycosaminoglycan (GAG) chains (e.g., chondroitin sulfate and keratan sulfate), which attract water molecules, create osmotic pressure in the ECM and retain water in the cartilage [89].

HA helps maintain the integrity of the pericellular matrix (PCM), a specialized layer of ECM that surrounds each chondrocyte and protects chondrocytes from mechanical stress [90]. HA interacts with aggrecan to form large, hydrated complexes that help improve cartilage elasticity. Hyaluronate–aggrecan complexes form the primary molecular mechanism providing cartilage elasticity and load-bearing capacity [34].

HA is a long-chain GAG composed of repeating disaccharide units that provide a strong and flexible structure for binding multiple aggregate molecules. The connection between HA and aggrecan is facilitated by a small molecule known as a linking protein that stabilizes the bond between HA and aggrecan, creating a strong and durable connection [91]. The aggrecan molecule has a core protein densely decorated with GAG side chains, mainly chondroitin sulfate and keratan sulfate. GAG chains have a high negative charge, attract water molecules, and create an osmotic environment that increases the hydration of the complex [92].

HA–aggrecan complexes interact with water to form a gel-like structure that fills the cartilage’s extracellular matrix (ECM). The gel-like matrix imparts unique viscoelastic properties to the cartilage, allowing it to compress and rebound. A high hydration level promotes the diffusion of nutrients to chondrocytes embedded in the cartilage, supporting their survival and function [93].

Highly hydrated HA–aggrecan complexes resist compression by generating internal swelling pressure. The negatively charged GAG chains on aggrecan repel each other and resist compression because of their electrostatic interactions [94]. By binding aggrecan to stable complexes, HA helps protect aggrecan from enzymatic degradation owing to the effects of enzymes typical of osteoarthritis, such as aggrecanases and matrix metalloproteinases (MMPs), which are elevated and degrade ECM components, including aggrecan [95].

The stability of the HA–aggrecan complex reduces the likelihood of cleavage and release of aggrecan from the ECM, thus preserving the cartilage matrix structure and function [92,93]. Large HA–aggrecan complexes evenly distribute mechanical loads in the cartilage. Forming a uniform and resilient matrix, these complexes help maintain the smooth cartilage surface necessary for efficient joint movement and reduce the local stress concentrations leading to cartilage wear. Through these molecular interactions, HA and aggrecan together form a resilient, hydrated matrix that enables the cartilage to absorb shock, resist compressive forces, and maintain its structural integrity [96]. Together, these molecular mechanisms contribute to the therapeutic effect of HA delivery in treating osteoarthritis.

Hyaluronic acid (HA) is a potent mediator of inflammation. This reduces the production of proinflammatory cytokines and joint inflammation. HA binds primarily to CD44 and the receptor for hyaluronan-mediated motility (RHAMM), which are expressed on the surface of synovial cells, chondrocytes, and immune cells [97,98]. The binding of HA to CD44 can inhibit the nuclear factor kappa-light-chain-enhancer (NF-κB) pathway, a master transcriptional regulator of inflammation [99,100]. Under inflammatory conditions, NF-κB translocates to the nucleus and promotes the expression of genes encoding proinflammatory cytokines, including interleukin-1β (IL-1β) and tumor necrotizing factor-α (TNF-α). Additionally, suppression of Toll-like receptor (TLR) activation occurs because HA interacts with Toll-like receptors (TLR-2 and TLR-4) on immune cells, which are frequently upregulated in OA and contribute to inflammation when activated [101]. By binding to these receptors, HA reduces the activation of TLR-mediated pathways that would otherwise trigger the production of inflammatory cytokines [102]. Another element is reducing reactive oxygen species (ROS), as HA exhibits antioxidant activity and thus modulates inflammation. By scavenging reactive oxygen species, HA reduces oxidative stress, which can otherwise activate inflammatory signaling pathways, including those that stimulate the release of IL-1β and TNF-α. HA also interacts with synovial membrane cells and chondrocytes, reducing cell sensitivity to proinflammatory cytokines by downregulating cytokine receptor expression on the cell surface, such as IL-1β and TNF-α [103,104].

HA’s role in promoting chondroprotection is critical for preventing and managing OA. HA stimulates the synthesis of endogenous hyaluronic acid and proteoglycans by chondrocytes, contributing to the maintenance and repair of the cartilage matrix [105,106]. This function of HA demonstrates its potential to protect and preserve the integrity of the cartilage matrix, which is a crucial aspect of OA management. This process contributes to the cartilage matrix’s maintenance, integrity, and repair. The activation of CD44 receptors on chondrocytes plays a significant role in this mechanism. Exogenous HA binds to CD44 receptors on the surface of chondrocytes, triggering intracellular signaling pathways that promote anabolic processes in these cells. CD44 is the primary receptor for HA in chondrocytes [107]. The binding of HA to CD44 activates the mitogen-activated protein kinase (MAPK)/extracellular signal-regulated kinase (ERK) signaling pathway, which is associated with increased chondrocyte proliferation and enhanced synthesis of ECM components [98,108]. The ERK pathway plays a crucial role in regulating the gene expression of matrix proteins, such as aggrecan and type II collagen. TGF-β signaling is enhanced under the influence of administered HA, which increases the activity of transforming growth factor beta (TGF-β) receptors on chondrocytes. TGF-β is an essential cytokine for cartilage health and stimulates the production of matrix components through the Smad signaling pathway, contributing to the maintenance and repair of the ECM. HA’s interaction with chondrocytes also reduces the expression of catabolic enzymes, such as matrix metalloproteinases (MMPs) and aggrecanase, which degrade ECM components in OA [109,110].

HA plays a protective role against chondrocyte apoptosis by modulating cell survival pathways, including the phosphoinositide three kinase (PI3K)/Akt pathway. HA interacts with cell surface receptors such as CD44 and RHAMM, activating the PI3K/Akt signaling pathway, a critical regulator of cell survival and apoptosis [98]. This activation leads to Akt phosphorylation, inhibiting pro-apoptotic signals by inactivating proteins like Bad, caspase-9, and Forkhead transcription factors (FOXO) [111]. Akt activation promotes cell survival by upregulating anti-apoptotic proteins, such as Bcl-2 and Bcl-xL, maintaining mitochondrial integrity and preventing cytochrome c release. HA can reduce oxidative stress in chondrocytes by enhancing the expression of antioxidant enzymes via the PI3K/Akt pathway [112]. Additionally, HA maintains the extracellular matrix (ECM), providing a supportive environment for chondrocytes. A well-maintained ECM can signal through integrins and other receptors to activate survival pathways, including PI3K/Akt [98].

HA acts as an antioxidant, scavenger of reactive oxygen species, and protects cartilage from oxidative stress-induced damage. This antioxidant function is crucial because ROS such as superoxide anions (O_2_^−^), hydroxyl radicals (OH-), and hydrogen peroxide (H_2_O_2_) accumulate in OA joints and contribute to cartilage degradation, inflammation, and chondrocyte apoptosis. HA’s ability to reduce oxidative stress provides a sense of security regarding its potential to protect cartilage from damage caused by OA [113].

The molecular mechanisms of antioxidant activity include direct uptake of ROS, inhibition of oxidative enzyme activity, regulation of antioxidant enzymes in chondrocytes, protection of cell membranes and DNA, reduction in ROS-mediated inflammatory signaling, and protection against apoptosis and aging [114]. The molecular structure of hyaluronic acid allows it to interact directly with ROS [115]. Hydroxyl groups (-OH) on HA can donate electrons to ROS, neutralize them, reduce their oxidative potential, and minimize oxidative stress on chondrocytes and the extracellular matrix (ECM) in cartilage. HA can inhibit the activity of enzymes, such as NADPH oxidase and xanthine oxidase, which generate ROS in response to inflammatory stimuli in the joint. By decreasing the activity of these enzymes, HA reduces ROS production at the cellular level, thereby reducing oxidative stress in the cartilage environment [116]. HA also stimulates intracellular signaling pathways that regulate antioxidant enzymes (mediated by CD44 binding). This interaction increases the activity and expression of antioxidant enzymes such as superoxide dismutase (SOD), catalase, and glutathione peroxidase in chondrocytes [117]. These enzymes play a key role in converting ROS into less harmful molecules, thereby protecting the cells from oxidative damage. ROS uptake by HA helps to protect chondrocyte cell membranes from lipid peroxidation, a destructive process in which ROS oxidizes lipids in cell membranes [103]. HA maintains the membrane integrity by preventing lipid peroxidation, which is essential for chondrocyte function. HA also reduces ROS-induced DNA damage, preserves genetic material in chondrocytes, and prevents apoptosis and mutations that could impair cartilage regeneration [112,118].

By scavenging ROS, HA indirectly reduces the activation of redox-sensitive inflammatory signaling pathways such as NF-κB and MAPK. ROS acts as secondary messengers in these pathways, promoting the expression of proinflammatory cytokines and matrix-degrading enzymes [103]. Chronic oxidative stress can induce chondrocyte apoptosis (cell death) and senescence (aging), processes that contribute to cartilage degeneration [19,119]. HA also interacts with pain receptors in the joint, attenuating nociceptive signaling and relieving pain. HA modulates pain in OA through several molecular mechanisms that affect nociceptive (pain) signaling in the joints. By interacting with specific pain receptors, particularly transient receptor vanilloid potential 1 (TRPV1) and acid-sensing ion channels (ASICs), and modulating inflammatory pathways, HA helps alleviate joint pain associated with OA [120,121]. These receptors are sensitive to changes in pH and inflammatory mediators, and their activation contributes to pain sensation. HA in the joint cavity creates a protective barrier around these receptors, reducing their exposure to inflammatory mediators and acidic byproducts generated by OA. HA has been shown to reduce the release of substance P, a neuropeptide involved in pain transmission and inflammation [122]. Substance P is released from nerve endings in response to joint damage and inflammation, sensitizing pain receptors and promoting the production of inflammatory cytokines [123]. The anti-inflammatory effects also include a reduction in the production of prostaglandin E2 (PGE2), a lipid mediator that sensitizes pain-sensing neurons (nociceptors) in response to inflammation [124]. HA can reduce the action of nerve growth factor (NGF), a protein involved in pain signaling and nociceptor sensitization. NGF levels are typically elevated in patients with OA and contribute to increased pain perception by increasing pain receptor sensitivity. Restoration of the joint viscoelastic properties of HA reduces the mechanical stress on the pain receptors in the joint [1].

The anti-inflammatory effect of hyaluronic acid also contributes to pain modulation by reducing the activation of redox-sensitive pain signaling pathways such as NF-κB and MAPK. In OA, these pathways are upregulated, increasing the production of proinflammatory cytokines and pain mediators that enhance nociceptive signaling. Thus, HA directly and indirectly modulates pain receptors, reduces the production of pain mediators, and improves the joint environment, collectively leading to reduced pain in patients with OA [125].

The binding of HA to CD44 activates the MAPK/ERK pathway for cell proliferation and matrix synthesis, which promotes chondrocyte proliferation and synthesis of ECM components, such as proteoglycans and collagen, by increasing the expression of matrix protein genes, thereby promoting anabolic processes necessary for cartilage maintenance and repair [108]. HA-CD44 binding also activates the phosphoinositide kinase 3 (PI3K)/Akt signaling pathway, which promotes cell survival and inhibits programmed cell death (apoptosis) [98,108]. The binding of HA to RHAMM and CD44 can activate the GTPase signaling Rho pathway, which regulates the cytoskeleton and is critical for chondrocyte and synoviocyte migration and repositioning within the joint, facilitating repair processes and helping cells migrate to areas of tissue damage [34,126].

HA may contribute to the maintenance of subchondral bone integrity by affecting the activity of osteoblasts and osteoclasts [127]. The molecular mechanisms underlying HA’s protective effects of HA on subchondral bone include interaction with osteoblast receptors to increase bone formation, inhibition of osteoclastogenesis through modulation of the RANKL/OPG pathway, reduction in inflammatory mediators affecting bone cells, modulation of Wnt/β-catenin signaling for bone remodeling, enhancement of osteoblast matrix production and mineralization, reduction in oxidative stress in bone cells, and support of angiogenesis [128,129,130]. HA, by binding to CD44 receptors on osteoblasts, activates signaling pathways that promote osteoblast proliferation, differentiation, and activity, such as the MAPK/ERK pathway, which is involved in osteoblast function and bone matrix production and inhibition of osteoclastogenesis through modulation of the RANKL/OPG pathway [131,132].

HA can affect the receptor activator of nuclear factor-kappa B ligand (RANKL)/osteoprotegerin (OPG) balance. RANKL promotes osteoclast formation and bone resorption, whereas OPG acts as a decoy receptor for RANKL and inhibits osteoclastogenesis [133]. The anti-inflammatory effect of HA on subchondral bone is achieved by reducing the levels of proinflammatory cytokines such as TNF-α and IL-1β, which stimulate osteoclast activity and inhibit osteoblast function [134].

The Wnt/β-catenin signaling pathway is central to regulating osteoblast activity and bone formation, which is Wnt/β-Catenin signaling for bone remodeling [135]. The activation of this pathway promotes osteoblast differentiation and reduces bone resorption.

In addition, the presence of HA in the joint environment promotes osteoblasts’ production of matrix proteins, including collagen and non-collagen proteins. It improves mineralization, bone density, and strength in the subchondral region [136].

HA can act as a scavenger of ROS, protecting osteoblasts from oxidative stress. HA preserves osteoblast function and reduces osteoclast-induced bone degradation, promoting healthy subchondral bone maintenance. HA also plays a role in supporting angiogenesis in the subchondral bone layer [137].

HA promotes balanced osteoblast and osteoclast activity through these mechanisms, thereby protecting subchondral bone from excessive degradation and abnormal remodeling associated with OA. This effect not only preserves the integrity of the bone but also indirectly protects the underlying cartilage, keeping the subchondral bone stable and resilient, reducing mechanical stress on the cartilage, and improving joint function.

HA is a long-chain GAG composed of repeating disaccharide units that provide a strong and flexible structure for binding multiple aggregate molecules [138]. A small molecule known as a linking protein facilitates the connection between HA and aggrecan, stabilizing their bond and creating a strong and durable connection [93].

The aggrecan molecule has a core protein densely decorated with GAG side chains, mainly chondroitin sulfate and keratan sulfate. GAG chains have a high negative charge, attract water molecules, and create an osmotic environment that increases complex hydration [93].

HA–aggrecan complexes interact with water to form a gel-like structure that fills the cartilage’s ECM [84,86]. The gel-like matrix imparts unique viscoelastic properties to the cartilage, allowing it to compress and rebound. The high hydration level promotes the diffusion of nutrients to chondrocytes embedded in the cartilage, supporting their survival and function.

Highly hydrated HA–aggrecan complexes resist compression by generating internal swelling pressure. The negatively charged GAG chains on aggrecan repel each other and resist compression because of their electrostatic interactions. This allows the cartilage to absorb impact and protect the underlying bone, providing joint stability and shock absorption during movement [93,94,96].

By binding aggrecan to stable complexes, HA helps protect aggrecan from enzymatic degradation. This is owing to the effects of enzymes typical of osteoarthritis, such as aggrecanases and matrix metalloproteinases (MMPs), which are elevated and degrade ECM components, including aggrecan [139].

The stability of the HA–aggrecan complex reduces the likelihood of cleavage and release of aggrecan from the ECM, thus preserving the cartilage matrix structure and function [140]. Large HA–aggrecan complexes evenly distribute mechanical loads in the cartilage. Forming a uniform and resilient matrix, these complexes help maintain the smooth cartilage surface necessary for efficient joint movement and reduce the local stress concentrations leading to cartilage wear. Together, these molecular mechanisms contribute to the therapeutic effect of HA delivery in treating osteoarthritis.

Over the past two decades, IAHA VS [39,41,43,44,68,141,142,143,144,145,146,147,148] has been widely adopted in clinical practice because it provides symptomatic relief and improves patients’ quality of life. The effectiveness of IAHA in treating osteoarthritis is confirmed by its dual role in the mechanical and biological processes occurring in the joint (Table 1) [149,150,151,152,153,154,155]. Mechanically, IAHAs replenish the loss of synovial fluid, thereby improving lubrication and shock absorption [34,43,105,152,155,156]. Biologically, IAHA exerts anti-inflammatory effects, modulates the local immune response [157,158], and stimulates endogenous HA production [43,159], contributing to joint homeostasis [7,43,60,160].

Considering the chronic nature of OA and the limitations of current treatments, IAHA VS is a valuable addition to the therapeutic armamentarium, especially for patients who do not respond adequately to first-line therapy. However, the efficacy of IAHA varies with its molecular weight, disease severity, and individual patient characteristics.

Studies are ongoing to refine the use of IAHA in clinical practice [33,35,38,161]. Numerous studies have investigated the efficacy of IAHA injections in OA treatment [33,40,44,55,56,63,149,162]. IAHA has provided symptomatic relief [45,50,54,55,65,142,149,163,164,165,166,167] and improved joint function [33,39,54,60,144,148,166,168] in many patients. The benefits of using HA vary between studies. Despite several debates regarding its effectiveness compared with other treatments, IAHA remains a widely used and essential option in nonsurgical OA management [33,34,35,68,169,170,171].

Because IAHA has become a routine method, the availability of new studies has decreased. The management of IAHA injections for OA is debated in the medical community.

Clinicians must navigate biological factors such as patient adherence, resource availability, and decisions regarding HA formulations (Table 2).

A critical consideration is choosing between low- and ultra-high-molecular-weight (UHMW) HA, which is influenced by patient characteristics, healthcare access, cost, and resource constraints. In resource-limited settings, clinicians may favor low-molecular-weight (LMW) HA owing to its lower cost and easier availability. UHMW HA may be preferred for patients with advanced OA requiring longer-lasting symptom relief, though it may be more expensive and less accessible [172,173].

Adherence to HA treatment protocols is critical. Some patients may discontinue treatment because of unmet expectations or perceived ineffectiveness, especially if they are unaware of the need for repeated injections. Educating patients about HA’s expected outcomes and the role of HA in managing OA symptoms is essential for improving adherence. Clinicians must make decisions based on resource availability because not all patients can access UHMW HA formulations, which are often more costly and may require specialized administration techniques [173]. Despite decades of use, the debate continues regarding its efficacy, safety, and administration protocols. HA injections mimic the viscoelastic properties of synovial fluid, improve joint lubrication, and modulate inflammatory responses [16,34,105,155]. However, discrepancies existed in the study’s outcomes. Randomized controlled trials have shown that some patients do not respond adequately to oral NSAIDs or physical therapy [33]. A network meta-analysis indicated that specific HA formulations may offer superior outcomes compared to placebo or other injectables such as corticosteroids [174]. Critical analysis revealed heterogeneity in patient selection, HA molecular weight, injection regimens, follow-up duration, and outcome measures, complicating the consensus on the effectiveness of HA.

Some studies have suggested that HA has superior results depending on the design and formulation [175]. Higher-molecular-weight HA preparations may offer substantial benefits for pain relief and functional improvement [160]. Despite over three decades of use [34], controversy persists regarding dosing schedules, preparation types, combination therapies, and safety concerns [33]. Advances have highlighted HA’s lubricating properties and biological activities of HA, which influence articular cartilage physiology [16,152,176]. HA shows potential disease-modifying effects by promoting extracellular matrix preservation/restoration, downregulating proinflammatory factors, and providing antinociceptive effects [32,105]. Studies have reinforced HA’s role in improving pain and joint function in knee OA (KOA) patients. HA injections significantly improved WOMAC pain and function scores compared to placebo at the monthly follow-up [174]. Patients receiving HA injections experience a significant reduction in pain and mobility, particularly in the early stages [177]. However, no significant difference in pain relief between HA and saline injections over 12 months has been reported [178], challenging HA’s universal effectiveness and suggesting its benefits may be more pronounced in certain patient subgroups or depending on specific HA formulations. These findings suggest HA may be particularly beneficial when incorporated early in treatment regimens as part of a comprehensive strategy to delay surgical interventions. HA-based hydrogel scaffolds have promising applications beyond joint diseases [179], specifically stem cell transplantation, highlighting HA’s versatility of HA but not providing direct evidence for its efficacy in traditional intra-articular injection contexts. HA hydrogels interact with immune cells, potentially impacting regenerative medicine applications, but do not directly translate to enhanced efficacy in conventional OA treatments [180].

Despite numerous studies investigating IAHA effects on joint conditions with varying patient populations, evidence quality remains mixed owing to methodological inconsistencies across trials. The heterogeneity among clinical trials complicates the establishment of definitive conclusions regarding HA’s role of HA in multimodal treatment regimens. Although it demonstrates potential as part of a comprehensive treatment strategy for OA through joint lubrication and possible cartilage health promotion [64,181,182], its standalone effectiveness remains controversial [183]. Clinical practice has shifted towards individualized patient care plans incorporating pharmacological and non-pharmacological interventions [184]. Future research should focus on elucidating optimal combinations of HA properties and establishing therapies to maximize patient outcomes.

### 3.4. Consideration of HA Formulations

Hyaluronans are the basic, non-cross-linked forms of hyaluronic acid, often derived from bacterial fermentation or animal sources, and vary primarily in molecular weight. Hylans, on the other hand, are chemically cross-linked variants of hyaluronic acid that offer increased viscosity and potentially longer intra-articular residence times compared to standard hyaluronans [185], and HYADD^®^4 compounds represent a further chemically modified class of hyaluronic acid, designed to improve viscoelastic properties, enhance biological activity, and optimize joint retention for more sustained therapeutic effects [186].

Low-molecular-weight (LMW) HA (<1 MDa) typically exhibits lower viscosity, providing less mechanical cushioning and shorter intra-articular residence times, which can lead to quicker clearance from the joint; in some cases, LMW fragments may be proinflammatory, though this varies by product and formulation. In contrast, high molecular weight (HMW) HA (~1–3 MDa) generally features higher viscosity, offering more robust mechanical protection and forming a thicker “coating” on cartilage surfaces; it also tends to remain in the joint longer, potentially yielding extended symptom relief and more pronounced anti-inflammatory effects by stabilizing the extracellular matrix and synovial environment.

The choice of LMW HA or UHMW HA is crucial when deciding on treatment. Studies have indicated that UHMW HA provides long-lasting pain relief and requires fewer injections, ideal for patients seeking longer intervals between treatments. LMW HA formulations are thought to have fewer immediate adverse effects and lower upfront costs, although they require frequent administration [60,100]. Clinicians must consider the clinical characteristics and practical factors, such as costs and patient access to follow-up care. Although clinical evidence supports the efficacy of both formulations, individualized treatment that suits patients’ financial and logistical situations is crucial.

The table (Table 3) summarizes several products that contain hyaluronic acid (HA). However, actual values may vary depending on the manufacturer’s formulas, newer research results, and updated labels.

In the table, molecular weight (MW) refers to the products from lower MW to cross-linked/high MW (“hylans”), each affecting viscosity and potential biological effects. Retention time indicates how long the injected material is expected to remain active in the joint, although individual patient factors (e.g., metabolism and activity level) can also influence this time. The application regimen can include weekly injections for several weeks; however, single-injection (one-shot) options are also available.

### 3.5. Inconsistencies in the Literature

The inconsistencies in HA VS efficacy may stem from variations in study design, patient populations, and HA formulations. Differences in molecular weight, cross-linking, and the number of injections significantly influenced the outcomes. UHMW HA formulations have shown more sustained pain relief than low-molecular-weight formulations. However, not all studies have accounted for these variations, leading to mixed results. Patient selection criteria vary widely across studies. Some trials included patients with mild OA, whereas others focused on more severe cases, influencing the overall effectiveness of HA. HA may be more effective in early-stage OA, where synovial fluid is less degraded, and the joint environment is more responsive to VS [198]. Total knee replacement (TKR) may be delayed in KOA patients with more HA courses [61].

The quality of evidence supporting HA use is mixed, ranging from moderate to low certainty across studies, questioning its role as a first-line treatment for contemporary OA. Given the current evidence landscape, indicating both HA’s potential benefits and limitations, clinicians may consider integrating newer therapies with traditional options rather than relying solely on HA VS.

UHMW HA is recommended for pain relief and functional improvement, particularly in moderate-to-severe OA [21,22,160]. Although individual studies of AHA show promising results, the overall landscape is characterized by variability in study design, patient demographics, dosing regimens, and follow-up periods. This inconsistency makes it difficult to establish HA as the definitive treatment for KOA and other joint disorders. Future studies should standardize the methodology and improve the study designs to obtain more reliable comparisons and conclusions regarding HA efficacy [199,200,201]. Rigorous trials with larger sample sizes and consistent outcome measures are essential to advance our understanding of IAHA therapies.

### 3.6. The Need for Standardized Protocols in HA Research

In assessing the role of IAHA in knee osteoarthritis management, a critical analysis revealed significant inconsistencies across various studies regarding its efficacy and application [202].

Chavda et al.’s [33] systematic review provided a comprehensive overview of IAHA’s safety and effectiveness. It concluded that IAHA can offer symptomatic relief for up to six months, with efficacy depending on the molecular weight and formulation type [33]. Combining IAHA with other treatments may enhance outcomes; however, further research is required. Most clinical practice guidelines (CPG) favor using IAHA as second-line therapy after conservative measures, contingent on individual patient profiles and responses to initial treatments [203]. A network meta-analysis of 137 studies ranked IAHA among interventions with modest efficacy but lacked long-term data [204].

Long-term pain control is associated with pharmacological treatments for OA beyond 12 months, highlighting the need for robust evidence before establishing IAHA as a standard treatment [205].

Although the literature supports IAHA use in OA management under specific conditions, substantial variability in clinical outcomes based on product formulation and injection protocols remains. Studies support IAHA as a viable treatment for patients who do not achieve adequate symptom relief from conservative measures [33,203]. However, many experts urge caution due to inconsistent evidence on its efficacy compared to other interventions [205,206,207]. The heterogeneity across trials complicates efforts to establish standardized protocols for their use.

There is an urgent need for well-designed RCTs with standardized methodologies to better delineate the role of IAHA within OA management strategies, particularly given the increasing interest in alternative therapies, which appear promising based on recent analyses [44,207,208,209].

### 3.7. Comparative Efficacy and Clinical Implications

When comparing LMW and UHMW HA formulations, evidence suggests that UHMW HA may offer longer-lasting symptom relief and require fewer injections, enhancing patient compliance and quality of life. LMW HA may be more suitable for patients who are sensitive to injection-related discomfort or those in the early OA stages. Despite the fact that both formulations were found to be clinically effective, UHMW HA showed superior pain reduction and functional improvement at six- and twelve-month follow-ups [179]. The choice between LMW and UHMW HA should be individualized based on patient-specific factors, including disease severity, comorbidities, treatment goals, and injection tolerance [210]. UHMW HAs, such as Hylan G-F 20 [35,67,167,211] are preferred for advanced OA because of their long-lasting effects and excellent joint lubrication properties. These formulations are recommended for patients requiring less frequent injections and seeking long-term relief. UHMW HA is often considered more effective in providing sustained pain relief and improving joint function [42].

Systematic reviews and meta-analyses over the past two decades have consistently demonstrated the superior efficacy of UHMW HA over LMW HA in managing OA. UHMW HA provided significant pain relief and functional improvement lasting up to six months [33]. In multiple studies [191], UHMW HA consistently yielded better pain reduction than LMW HA and revealed significant differences in clinical outcomes based on the molecular weight and source of HA used in the treatment protocols.

Among the injectable treatments for OA, only UHMW HA surpassed the minimal clinically essential difference thresholds for pain relief and functional improvement [212]. This underscores the importance of selecting appropriate HA formulations based on their molecular weight. As noted by Aggarwal and Sempowski [193], UHMW HA is considered more effective than LMW formulations in improving pain and function in patients with OA.

A systematic review of HOA supports the idea that UHMW HA provides superior outcomes without increased adverse effects compared to other molecular weights [173]. High-molecular-weight HAs are preferred over low-molecular-weight variants for KOA management because of their better efficacy in reducing pain and improving function while being cost-effective compared to traditional therapies. Although some studies suggest limited benefits of postsurgical HA injections [163], the evidence favors their use as a conservative treatment before surgery. Systematic reviews and meta-analyses have shown that UHMW HA is more practical for managing OA symptoms [67,203,212]. Indications and patient selection are crucial. HA administration is generally recommended for patients with mild-to-moderate OA, particularly KOA, who do not respond adequately to first-line treatment. IAHA mainly benefits patients who want to delay or avoid surgery such as total knee arthroplasty (TKA) [22]. LMW HA is often preferred in patients requiring frequent injections because of its short half-life and is typically administered in three to five weekly injections. Clinical benefits can last several months; however, repeated cycles are often necessary [147]. UHMW HA has a longer duration of action and is used in patients who require fewer injections. These formulations have better viscoelastic properties, potentially resulting in more significant and longer-lasting symptom relief [165,213]. Some patients may respond better to LMW HA, suggesting individualized treatment plans are needed [42]. Both are generally safe, with the most common side effects being local injection-site reactions. UHMW HA formulations have a slightly higher incidence of side effects, possibly because of the more viscous nature of the product [22]. Shared decision-making between medical specialists and patients is essential for optimal treatment outcomes.

Evidence from clinical trials has shown that HA injections lead to significantly better outcomes than placebo or nonsteroidal anti-inflammatory drugs (NSAIDs). The current literature supports HA’s efficacy in treating OA symptoms and suggests its potential disease-modifying effects (Table 4), but clinical practice guidelines remain inconsistent [30]. HA is considered as a second-line option owing to its safety profile and potential to delay the need for TKA.

Individualized care and tailored treatment approaches considering disease severity, patient age, activity level, and prior treatment response remain essential for optimizing outcomes.

Implications for clinical practice include incorporating IAHA VS into treatment regimens for moderate to severe OA, which may significantly benefit patients. Clinicians should consider patient-specific factors, including disease severity, prior treatment response, and individual health profiles when implementing IAHA [210,214,215].

Acknowledging the current review limitations is essential to ensuring the accurate interpretation and generality of findings regarding IAHA in OA. The limitations include significant heterogeneity in design, sample size, follow-up duration, outcome measures, and patient population, making direct comparisons difficult. Variability in HA administration protocols further complicates the efficacy assessment. The inclusion criteria often varied, introducing a potential bias. The placebo effect and short follow-up may limit understanding of its long-term benefits. Although short-term benefits are well documented, the lack of long-term data beyond two years remains a fundamental limitation. Future research should focus on standardizing treatment protocols, investigating cost-effectiveness in preventing surgical intervention, and evaluating combination therapies involving HA and other treatments, such as platelet-rich plasma (PRP), to enhance patient outcomes [200,216]. Further studies addressing these issues will help resolve inconsistencies in clinical practice guidelines and improve the standardization of HA treatment protocols, leading to more reliable patient outcomes.

Current research on IAHA(HA) therapy faces limitations due to variability in study design and outcomes across conditions such as knee OA. Inconsistency in patient populations, treatment protocols, and follow-up durations hinders firm conclusions regarding the efficacy of HA. Costa et al. found that platelet-rich plasma (PRP) may offer pain relief similar to HA, but the evidence quality was low owing to study bias [199]. Garcia et al. [200] reported no significant difference in pain reduction between PRP and HA groups. These inconsistencies highlight the need for standardized research methodologies to evaluate the clinical benefits of HA. Xavier et al. [217] noted that innovative treatments like exosome-laden scaffolds face challenges such as rapid clearance rates and inconsistent dosing regimens. Cui et al. [218] found that patients receiving HA injections had a higher risk of knee arthroplasty than those receiving corticosteroids. Chen et al. [219] explored traditional Chinese medicine (TCM) as an alternative to HA for OA management, but clinical heterogeneity complicated direct result comparisons.

Research on newer therapies, such as adipose-derived stem cell therapy, has shown promise. However, Kim et al.’s [201] review noted inconclusive evidence due to the variability in the study methodology. Riley et al. [220] concluded that no injection-based therapies for base-of-thumb osteoarthritis, including HA, demonstrated superiority over placebo, emphasizing the need for more high-quality randomized controlled trials.

### 3.8. Future Directions

Further research is needed to optimize IAHA treatment protocols to enhance outcomes for specific patient populations, particularly when exploring combination therapies such as IAHA with PRP. Refining the patient selection criteria will also ensure that IAHA is used effectively by those most likely to benefit from it.

Future research should focus on larger, more diverse populations with standardized treatment protocols, extended follow-up periods, and consistent and objective outcome measures [50,202,203,221,222,223,224]. Comparative studies are also needed to assess HA’s relative effectiveness and cost-effectiveness compared with alternative treatments. Studies should examine the effects of different molecular weights and formulations on outcomes and the interaction between HA and other conservative treatments, such as physical therapy or new pharmacological agents.

The therapeutic usefulness of IAHA can be optimized toward precision medicine by tailoring treatment to individual patient phenotypes, such as OA with inflammation dominance, mechanical overload, or post-traumatic OA. Incorporating biomarkers, imaging characteristics, and disease stages into treatment decisions could guide the choice of molecular weight, formulation type, and injection schedule. Such a stratified approach holds promise for maximizing clinical outcomes and advancing IAHA toward a precision medicine model for the treatment of osteoarthritis.

## 4. Conclusions

Intra-articular hyaluronic acid (IAHA) represents a versatile and evolving therapy for osteoarthritis, combining mechanical relief with biological modulation. Future directions include exploring personalized treatments based on patient-specific factors to enhance therapeutic outcomes. IAHA is a versatile therapeutic option for OA, offering symptomatic relief and potential disease-modifying benefits. Its multifaceted mechanisms, including anti-inflammatory, chondroprotective, and pain-modulating effects, underscore its role in personalized OA management. Stage-specific applications based on molecular weights and biomarker-driven approaches have advanced precision medicine. This review analyzes the efficacy, safety, and clinical use of IAHA for treating OA. Results indicate that IAHA effectively reduces pain and improves joint function in OA, particularly in the knees. IAHA injections provide significant short- and medium-term pain relief and improved function. Although data beyond two years are limited, repeated treatments have shown durable benefits in several studies. Long-term studies are necessary to confirm these findings. HMW HA formulations offer superior symptom relief and longer-lasting effects than low-molecular-weight formulations. Future studies should optimize the treatment protocols and identify ideal HA formulations for patient profiles. IAHA remains a safe treatment option, with a low incidence of adverse events, primarily local injection site reactions. The minimal occurrence of systemic adverse events underscores a favorable safety profile compared to alternative treatments. IAHA remains viable due to its established safety, ease of administration, and patient satisfaction. Comparative studies are required to assess the long-term efficacy and cost-effectiveness of IAHA compared with newer treatments. Future research should focus on long-term efficacy studies, direct comparisons between IAHA and other treatments, and studies that optimize dosing schedules and explore personalized treatment protocols. IAHA remains integral to osteoarthritis treatment, particularly for patients seeking nonsurgical options. Its efficacy and safety profile support its continued use, although future studies are necessary to elucidate its role in new therapies and optimize treatment regimens for different patient populations.

IAHA is a multifaceted, relatively safe, and patient-friendly treatment that offers meaningful pain relief and improved function, particularly in knee osteoarthritis.

Higher-molecular-weight formulations may provide superior, longer-lasting benefits, yet further research is needed to optimize dosing regimens and compare IAHA’s long-term efficacy with emerging alternatives.

Personalizing IAHA therapy based on patient-specific biomarkers and disease characteristics ultimately enhances outcomes and solidifies its role in nonsurgical osteoarthritis management.

Key Takeaways for Clinicians:

IAHA is a well-tolerated, multifaceted therapy offering symptomatic relief and potential disease-modifying effects via anti-inflammatory, antioxidant, and chondroprotective mechanisms.

Molecular weight matters: Low-molecular-weight (LMW) HA is best suited for early-stage OA and resource-limited settings. Ultra-high-molecular-weight (UHMW) HA demonstrates longer-lasting symptom relief and may benefit patients with advanced OA.

Personalized treatment strategies based on OA phenotypes, disease stage, and patient-specific biomarkers can improve therapeutic outcomes.

Adherence to injection protocols and patient education on expected outcomes are essential to maximizing IAHA’s clinical benefits.

IAHA should be considered in multimodal treatment strategies, particularly for patients aiming to delay or avoid surgical intervention like total joint arthroplasty.

## Figures and Tables

**Table 1:** Summary of HA’s molecular mechanisms in OA.

Mechanism	Key Molecular Interactions	Signaling Pathways Involved	Functional Outcome
Synovial Fluid Viscosity	-HA supplementation increases the concentration and size of HA molecules in the synovial fluid	-Primarily physical effects (no direct signaling)	-Improved joint lubrication, shock absorption, and smoother movement
Chondroprotection	-Formation of HA–aggrecan complexes via linking proteins	-Activation of CD44 leading to MAPK/ERK and PI3K/Akt pathways	-Enhanced ECM synthesis, cartilage repair, and protection against enzymatic degradation
Anti-inflammatory Effects	-HA binding to receptors (CD44, RHAMM, TLRs)	-Inhibition of NF-κB and suppression of TLR-mediated pathways	-Reduced production of proinflammatory cytokines (e.g., IL-1β, TNF-α) and decreased joint inflammation
Antioxidant Activity	-Direct scavenging of reactive oxygen species (ROS) via HA’s hydroxyl groups	-Upregulation of antioxidant enzymes via CD44-mediated signaling	-Reduced oxidative stress; protection of chondrocytes and preservation of ECM integrity
Bone Remodeling	-Modulation of osteoblast and osteoclast activities	-Regulation via RANKL/OPG balance and Wnt/β-catenin signaling	-Maintenance of subchondral bone integrity and balanced bone remodeling

**Table 2:** Comparison of HA formulations used in IAHA for OA treatment.

HA Formulation	Key Characteristics	Clinical Efficacy	Cost/Accessibility Considerations
Low-Molecular-Weight (LMW) HA	-Smaller size, lower viscosity	-Shorter duration of effect; effective in mild OA	-Generally less expensive and more readily available
High-Molecular-Weight (HMW) HA	-Larger size, higher viscosity	-Longer lasting effect; improved joint function	-Higher cost; production is more complex
Ultra-High-Molecular-Weight (UHMW) HA	-Largest molecules, very high viscosity	-Longest duration of effect; effective in advanced OA	-Most expensive; limited availability; may require specialized administration

**Table 3:** Summary of several hyaluronic acid (HA) products.

Product (Example Brand Name)	Approx. Molecular Weight Range	Estimated Retention	Typical Application Scheme
Sodium Hyaluronate (Hyalgan) [187]	500–730 kDa	1–2 weeks	1 injection/week for 5 weeks
Sodium Hyaluronate (Supartz) [188]	620–1170 kDa	1–2 weeks	1 injection/week for 5 weeks
Sodium Hyaluronate (GenVisc 850) [189]	620–1170 kDa	1–2 weeks	1 injection/week for 5 weeks
Hylan G-F 20 (Synvisc) [190]	~6000 kDa (6 MDa)	2–4 weeks	1 injection/week for 3 weeks
High MW HA (Orthovisc) [191]	1.0–2.9 MDa	1–3 months	1 injection/week for 3–4 weeks
Cross-linked HA (Monovisc) [192]	2.5+ MDa	Up to 4 months	Single injection
Non-animal stabilized HA(Durolane) [193]	~3.0 MDa	Up to 4 months	Single injection
1% Sodium Hyaluronate (Euflexxa) [194]	~2.4–3.6 MDa	1–2 months	1 injection/week for 3 weeks
HA chemically modified (Gelsyn-3) [195]	~1.1 MDa	1–3 months	1 injection/week for 3 weeks
Sodium Hyaluronate (Visco-3) [196]	620–1170 kDa	1–3 months	1 injection/week for 3 weeks
Hyadd^®^4 (Hymovis^®^) [197]	500–730 kD	Detected in the joint space for at least 25 days post-injection	two intra-articular injections of 3 mL each, administered one week apart

**Table 4:** Summary of clinical study outcomes for IAHA in osteoarthritis.

Patient Population	HA Formulation and Dosage	Outcome Measures	Key Findings
Moderate OA	HMW HA, series of 3 injections	WOMAC scores, pain reduction	Statistically significant reduction in pain and improved joint function
Mild OA	LMW HA, single injection	Range of motion, patient self-assessment	Short-term improvement; effect lasted for several weeks
Advanced OA	UHMW HA, series of 5 injections	Pain scale changes, joint function improvement, duration of effect	Long-lasting improvement; superior efficacy in advanced OA group
Various age groups and OA severities	Various HA formulations; comparison with placebo and corticosteroids	Pain scales, joint function, duration of effect	Some HA formulations showed higher efficacy than placebo; outcomes varied with dosage and formulation

## Data Availability

No new data were created for this study.

## Abbreviations

ADL	Activities of Daily Living
ASICs	Acid-Sensing Ion Channels
CPG	Clinical Practice Guidelines
ECM	Extracellular Matrix
ERK	Extracellular Signal-Regulated Kinase
GAG	Glycosaminoglycan
HA	Hyaluronic Acid
HAS1	Hyaluronan Synthase 1
HMW	High Molecular Weight
HOA	Hip Osteoarthritis
IAHA	Intra-Articular Hyaluronic Acid
IL-1β	Interleukin One Beta
KOA	Knee Osteoarthritis
LMW	Low Molecular Weight
MAPK	Mitogen-Activated Protein Kinase
MMPs	Matrix Metalloproteinases
NADP	Nicotinamide Adenine Dinucleotide Phosphate
NF-κB	Nuclear Factor Kappa-Light-Chain-Enhancer
OA	Osteoarthritis
OPG	Osteoprotegerin
PCM	Pericellular Matrix
PGE2	Prostaglandin E2
PI3K	Phosphoinositide Three Kinase
RCT	Randomized Clinical Trial
RHAMM	Receptor for Hyaluronan-Mediated Motility
RANKL	Receptor Activator of Nuclear Factor Kappa-B Ligand
ROS	Reactive Oxygen Species
TGF-β	Transforming Growth Factor Beta
TKA	Total Knee Arthroplasty
TKA	Total Knee Replacement
TLR	Toll-Like Receptor
TNF-α	Tumor Necrosis Factor Alpha
TRPV1	Transient Receptor Vanilloid Potential 1
UDP	Uridine Diphosphate
UDP-GlcUA	UDP-α-D-Glucuronate
UHMW	Ultra High Molecular Weight
VS	Viscosupplementation
WHO	World Health Organization
